# Alcohol craving and levels of suppression of depression, anxiety and anger in patients before and after alcohol dependence treatment in the form of a day ward

**DOI:** 10.3389/fpsyt.2026.1722260

**Published:** 2026-03-04

**Authors:** Marta Giezek, Aleksandra Szylińska, Paulina Zabielska, Beata Karakiewicz

**Affiliations:** 1 Subdepartment of Social Medicine and Public Health, Department of Social Medicine, Pomeranian Medical University in Szczecin, Szczecin, Poland; 2 Department of Cardiac Surgery, Pomeranian Medical University in Szczecin, Szczecin, Poland

**Keywords:** alcohol craving, alcohol dependence, anger, anxiety, depression, emotions

## Abstract

**Introduction:**

Craving for alcohol is an intense desire to drink alcohol, leading to an upsetting of the previous balance through the appearance of anxiety, cognitive dissonance and irritability. This desire, which varies individually, is closely linked to changes in the central nervous system and affects memory, attention, perception, biological functions and decision-making. It is associated with a strong motivation to drink and obsessive thoughts about contact with alcohol to reduce the discomfort experienced. The aim of this study is to investigate whether patients experience a reduction in alcohol craving and a change in anxiety and anger suppression after completing a primary treatment programme for alcohol dependence.

**Material and methods:**

The research was carried out by using the diagnostic survey method. In order to identify problems, the following were used: 1) CECS - Emotional Control Scale; 2) PACS (Penn Alcohol Craving Scale) - Polish adaptation of the Penn Alcohol Craving Scale (PACS). Changes in somatic, mental, social, occupational, family and social functioning were examined using a survey questionnaire of our own design. The study was conducted in 2024 at an Addiction Therapy Centre in north-western Poland. Patients of the Centre completed the toolkit during the first and the last (eighth) week of primary therapy.

**Results:**

A total of 156 patients participated in the study, from which a total of 154 respondents were qualified. A significant decrease in the PACS scale question total was observed (p<0.001). Before therapy, the median PACS scale score was 9 (4.0-16.0) and after therapy it was 4 (1.0-8.0). The suppression of depressive feelings was observed to increase with increasing alcohol craving, whereas after therapy, the ability to control anger was shown to increase with decreasing intensity of alcohol craving.

**Conclusions:**

Alcohol dependence therapy in the form of a day ward significantly reduces the severity of alcohol craving and increases the competence to recognise and name negative emotions, as well as to talk about and express them in an acceptable way. The most significant factor influencing the level of expression of adverse emotions is work activity.

## 1 Introduction

Alcohol is legal and still the most popular and available psychoactive substance in the world. Its consumption can lead to addiction and to irreversible health consequences. Alcohol abuse is responsible for 5.3% of all deaths worldwide and 1% of the global burden of disease and injury. Alcohol is an established carcinogen and its consumption increases the risk of several cancers, including breast, liver, head and neck, oesophageal and colorectal cancers. Recent World Health Organisation (WHO) recommendations make it clear that no amount of alcohol is safe for health, and there is no form of alcohol consumption that is risk-free (1–5).

The diagnosis of alcohol use disorder (AUD) is based on the identification of a persistent, problematic pattern of alcohol consumption leading to clinically significant distress or impairment in functioning, manifested by the presence of at least two symptoms within the past 12 months (6). These symptoms primarily include impaired control over drinking (such as consuming alcohol in larger amounts or over a longer period than intended, persistent unsuccessful efforts to cut down or control use, excessive preoccupation with alcohol, and subjectively experienced craving), impairment in social and occupational functioning (failure to fulfill major role obligations, interpersonal conflicts, and withdrawal from important activities), as well as continued alcohol use despite negative consequences and in situations involving increased risk.

The clinical picture also includes pharmacological criteria, such as the development of tolerance and the occurrence of withdrawal symptoms or the use of alcohol to relieve or avoid them (6, 7). The severity of AUD is determined by the number of criteria met, distinguishing mild (2–3 symptoms), moderate (4–5 symptoms), and severe (≥6 symptoms) forms. In this context, alcohol craving is of particular importance, as it constitutes not only one of the diagnostic criteria but also plays a significant role in the etiology of the disorder, its treatment, and the maintenance of abstinence (8).

Początek formularza.

Dół formularza.

### 1.1 Alcohol craving in the light of definitions

There are many definitions of alcohol craving. It is most commonly described as an uncontrollable craving for the substance (9) or as a psychological state that compels one to relapse into alcohol (10). According to the International Statistical Classification of Diseases and Health Problems ICD-10, alcohol craving is a strong desire or feeling of compulsion to take the substance (11). Alcohol craving is also defined as intense thoughts about alcohol, a strong need to feel pleasure from alcohol use or compulsive desire (12).

An extended and multifaceted definition is to describe alcohol craving as an intense desire to drink alcohol, leading to an imbalance of the previous balance through the appearance of anxiety, cognitive dissonance and irritability. This desire, which varies individually, is closely linked to changes in the central nervous system and affects memory (recall of information), attention, perception, biological functions (heart rate, blood pressure, saliva secretion) and decision-making. It is associated with a strong motivation to drink and obsessive thoughts about contact with a psychoactive substance to reduce the discomfort experienced (13). There are four groups of causes of alcohol craving and three groups of symptoms (**Table 1**).

**Table 1:** Summary of causes and symptoms of alcohol craving.

Causes		Symptoms	
Physiological	Neurochemical disorders: Prolonged drinking of alcohol can lead to changes in brain function, particularly in areas responsible for reward and pleasure, resulting in increased cravings for alcohol. Physical dependence: Regular alcohol consumption causes the body to adapt to the presence of alcohol, which can lead to physical dependence and alcohol craving as a reaction to the absence of the substance.	Physical	Trembling of the body especially the hands especially noticeable after prolonged periods without alcohol. Sleep disturbances: difficulty falling asleep, disturbing sleep or frequent waking can be a signal of alcohol craving. Excessive sweating and nausea: These symptoms often occur as a reaction of the body to the absence of alcohol.
Psychological	Coping with stress: People who use alcohol as a way to cope with stress may experience alcohol cravings in tension-provoking situations. Emotional problems: Negative emotions such as sadness, loneliness or low self-esteem can lead to seeking solace in alcohol.	Psychological	Obsessive thoughts about alcohol: Constantly thinking about drinking, planning the next occasion to drink alcohol. Nervousness and irritability: Changes in behaviour that occur when a person cannot consume alcohol.
Socio-cultural	The influence of an environment where drinking is the social norm can promote the development of alcohol craving, especially in groups with a high tolerance for alcohol consumption.	Behavioural changes	Neglecting responsibilities: Neglecting work, family or other commitments in favour of drinking. Avoidance of non-drinking situations: Choosing occasions and social gatherings for the availability of alcohol.
Genetic	Genetics may play a role in predisposition to alcohol dependence, which affects the risk of alcohol craving.		

Source: own elaboration based on (8, 14, 15).

Recent empirical studies demonstrate that alcohol craving is functionally linked to the regulation of negative emotional states, rather than being merely a response to alcohol-related cues. Clinical research on alcohol use disorder (AUD) indicates that fluctuations in subjective alcohol craving during treatment closely co-occur with changes in depression and anxiety, suggesting the presence of a shared affective mechanism that sustains the motivation to drink (16). Results from experimental studies further confirm that the induction of negative emotions leads to an increase in alcohol craving primarily among individuals who experience difficulties in emotion regulation, whereas brief interventions aimed at strengthening regulatory capacities significantly attenuate this effect (17). Research on emotional stress induction indicates that coping strategies—particularly the tendency to “drink for relief”—moderate the strength of the association between negative affect and alcohol craving, suggesting that it is not alcohol-related cues per se, but rather the use of alcohol as a tool for emotion regulation that drives the subjective desire to drink (18). Under naturalistic conditions, this relationship is supported by diary-based studies showing that momentary mood fluctuations and drinking motives oriented toward tension reduction predict increases in subjective alcohol craving in real time, even before alcohol consumption occurs (19). Depressed mood, anxiety, and anger constitute particularly strong triggers of drinking motivation. Consistent with this, experimental induction of anger leads to an increase in subjective alcohol craving, indicating a key role of affective arousal in the activation of addiction-related schemas (20).Taken together, the results provide a basis for a model in which the level of experienced alcohol craving reflects the current need to suppress or regulate negative emotional states.Based on the above findings, the hypothesis was formulated that effective treatment of alcohol dependence should lead not only to a reduction in alcohol craving itself, but also to a weakening of its associations with negative emotional states, particularly depression, anxiety, and anger.

The aim was to investigate whether patients experience a reduction in alcohol craving and a change in anxiety and anger suppression after completing a primary treatment programme for alcohol dependence.

## 2 Methods

### 2.1 Research questions

The research process sought to answer relevant questions from the point of view of change in the functioning of the alcohol-dependent person after completion of primary therapy.

1. What changes occur in the perceived craving for alcohol in patients before and after primary therapy in a day ward setting?
2. Does the level of severity of alcohol craving cause changes in the ability to respond to experiencing depression, anxiety and anger in patients before and after primary therapy?
3. What determinants change the level of suppression of depression, anxiety and anger in patients before and after primary therapy?

### 2.2 Study procedure

This article is being carried out as part of a research project entitled: ‘Assessment of the level of change in functioning of patients with alcohol dependence participating in primary therapy in a day ward setting’. Treatment in a day ward is a guaranteed health service reimbursed under a contract signed with the Payer – the National Health Fund (NFZ) and is free of charge for patients. It lasts for eight weeks, from Monday to Friday from 8 a.m. to 1 p.m. The therapy is an open group, which means that new participants join every week. The variability of patients means that participants can be at completely different levels from each other. This has its advantages – someone who is just starting therapy can benefit from the experience of patients who are just completing their therapy cycle. Patients in therapy benefit from the consultation of a psychiatrist. If necessary, it is possible to obtain a medical release for incapacity for the duration of the therapy. Patients are provided with one meal. Participants in this form of therapy can benefit from the intensity of psychotherapeutic measures comparable to inpatient units, while maintaining contact with loved ones and making ongoing adjustments to their behaviour leading to changes resulting in sober functioning. The programme of activities is multidimensional, tailored, following the needs of the patients based on the integrative stream. It includes psycho-education, workshops, relaxation techniques, training in coping with stress, anxiety, shame and anger, communication training and assertiveness training. All groups are run by specialists in addiction psychotherapy. Basic therapy in the day ward prepares patients to continue therapy in an in-depth form in the outpatient setting of the Addiction Therapy Clinic.

The study was conducted in 2024 at an Addiction Therapy Centre in north-western Poland, in three simultaneously ongoing therapy groups in the form of a day ward, in which the programme was led by three different addiction psychotherapists. Patients of the Centre completed a toolkit on the first day of starting therapy. Each participant was given information about the purpose and conduct of the study, along with a written assurance that they could opt out at any stage of the research procedure, without having to provide information about the reasons for this decision. The next study with the identical toolkit took place on the first day of the eighth (last) week of the therapy. Each patient was informed about the results of the tests during an individual interview in the presence of an addiction psychotherapist. This provided an opportunity to analyse the results in terms of changes in functioning, to identify areas of progression and areas for further therapeutic work. The final week of group work provided an opportunity for therapists to reinforce patients in the context of the results obtained.

The research was carried out by using the diagnostic survey method. In order to identify problems the following scales were used:

1. CECS – Control of Emotions Scale – Polish adaptation by Zygfryd Juczyński (21). It is used to measure subjective control of anger, anxiety and depression in difficult situations. The scale is designed to test healthy and ill adults. It consists of 21 statements that allow for a total score relating to emotion control and three subscales relating to anger control, depression control and anxiety control. Wyższe wyniki wskazują na większą deklarowaną tendencję do tłumienia i powstrzymywania ekspresji danej emocji, natomiast niższe wyniki odzwierciedlają mniejszy stopień jej hamowania i większą skłonność do ujawniania lub przeżywania emocji w sposób otwarty. Skala nie mierzy skuteczności ani adaptacyjności regulacji emocji, lecz poziom subiektywnie doświadczanej kontroli ich ekspresji (21).
2. PACS *(Penn Alcohol Craving* Scale*)* – Polish adaptation of the Penn Alcohol Craving Scale (PACS) (22, 23). It consists of five questions, each with seven answers ranging from 0 to 6. Questions 1 to 3 relate to the frequency, intensity and duration of craving, question 4 measures the ability to resist the temptation to use alcohol when there is an opportunity to drink, and question 5 is responsible for measuring the degree of overall craving for alcohol over the past week. The calculation of the results is based on the summation of the values of the answers, which range from 0 to 30. In the interpretation of the results, three ranges are indicated, and so it is estimated that the range 0-3 indicates a low severity of craving, the range 4-9 an average severity, and the results above 10 indicate a high severity of craving for alcohol (24). The performed adaptation of the Scale indicates that it has very good psychometric properties. It can be used for research and therapeutic purposes. The Cronbach’s alpha coefficient is 0.89 (23).

Changes in somatic, psychological, social, occupational, family and social functioning were examined using a self-constructed questionnaire survey. Respondents performed a self-assessment of their level of functioning in each of the specified domains using a five-point Likert scale, ranging from 1 (“worst”) to 5 (“best”). The questionnaire also included questions on sociodemographic characteristics, housing and financial situation, as well as information on the duration of abstinence and previous forms of alcohol dependence treatment.

### 2.3 Participants

A total of 156 participants were included in the study, from which a total of 154 respondents were qualified, including: 50 (32.47%) women and 104 (67.53%) men. The age of the respondents ranged from 43.5 ± 11.7 years. Respondents mostly lived in urban areas 140 (90.91%) vs rural areas 14 (9.09%) people. Most had secondary education 48 (31.17%) and least had primary education 5 (3.25%). There were 91 (59.48%) people who were economically active and 62 (40.52%) people who were inactive. For 47 (30.71%) of the respondents, this is another therapy. Respondents included in the study are residents of north-western Poland. Detailed characteristics of the surveyed persons are presented in **Table 2**.

**Table 2:** Characteristics of the surveyed group.

		n	Percent
Gender	female	50	32, 47
	male	104	67, 53
Age	18-35 years	40	26, 32
	36-60 years	95	62, 50
	≥60 years	17	11, 18
Place of origin	urban	140	90, 91
	rural	14	9, 09
Education	primary	5	3, 25
	lower secondary	10	6, 49
	vocational	46	29, 87
	secondary	48	31, 17
	higher	45	29, 22
Marital status	single	70	46, 05
	in a relationship	82	53, 95
Number of children	0	62	40, 79
	1	37	24, 34
	2	43	28, 29
	3	9	5, 92
	5	1	0, 66
Professional activity	yes	91	59, 48
	no	62	40, 52
Type of work performed	physical	73	58, 40
	white-collar	33	26, 40
	retired	14	11, 20
	on pension	5	4, 00
Financial situation	very good	14	9, 15
	good	53	34, 64
	average	58	37, 91
	bad	20	13, 07
	very bad	8	5, 23
Housing conditions	very good	32	20, 92
	good	78	50, 98
	average	34	22, 22
	bad	8	5, 23
	very bad	1	0, 65
Previous treatment for alcohol addiction	no	106	69, 28
	yes, 1 to 2 years previously	23	15, 03
	yes, from 3 to 5 years before	9	5, 88
	yes, 6 years ago and before	15	9, 80

n-number of patients.

### 2.4 Statistical analysis

Statistical analysis was performed using the licensed software Statistica 13.0 (StatSoft, Inc. Tulsa, OK, USA). The Shapiro-Wilk test was used to assess the normality of the distribution of the study variables. Medians and quartiles, as well as counts and percentages, were mainly used to present group characteristics. The evaluation of differences between the study group and control group was performed using the Mann-Whitney U test. The Wilcoxon test was used for analysis before and after therapy For the analysis of qualitative data, the X2 test was used; if there was a low abundance in a subgroup, the Yates correction was applied. Correlation analysis was performed using Spearman’s rank correlation. A significance level of p ≤ 0.05 was adopted.

## 3 Results

**Figure 1:**
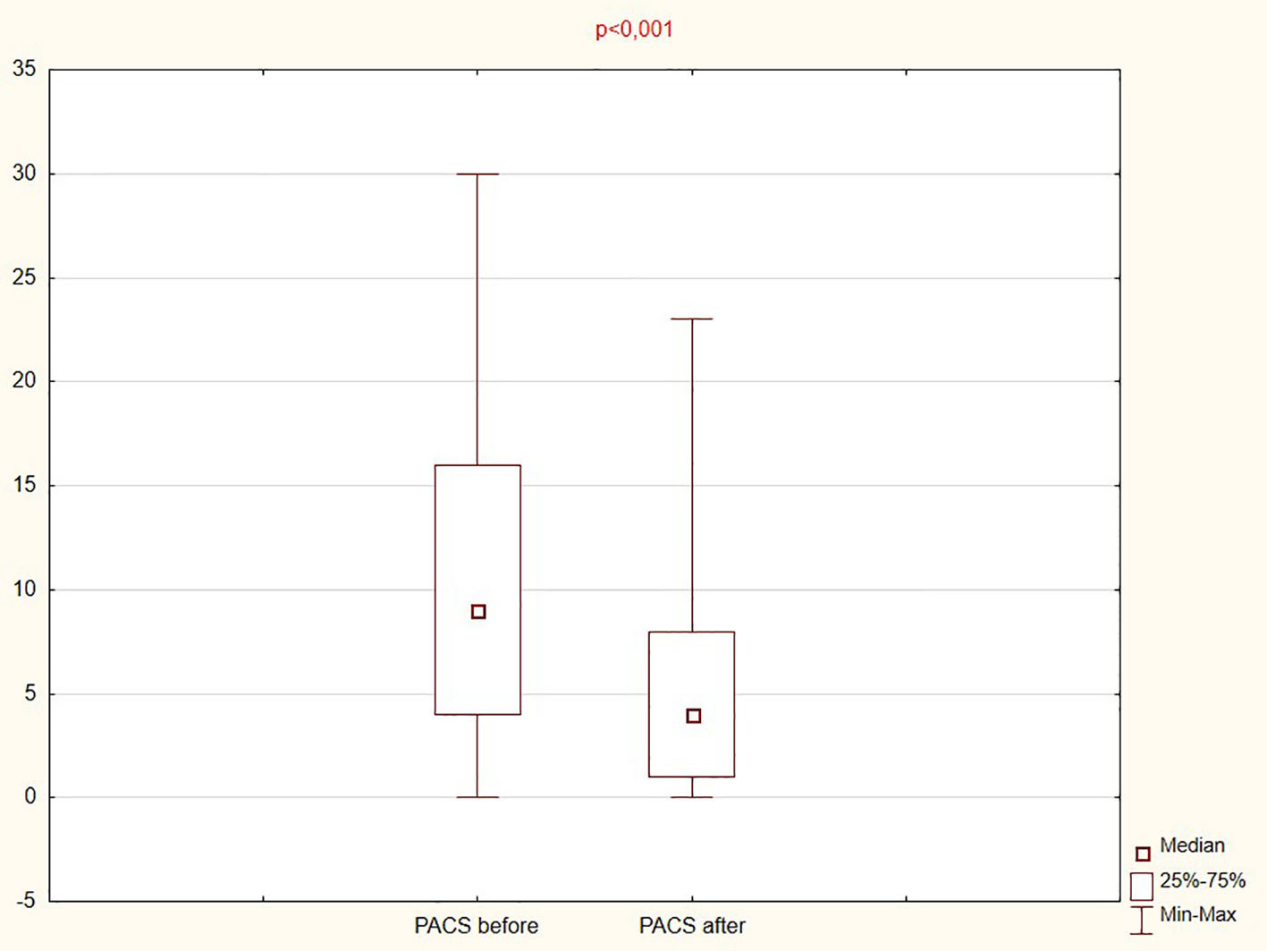
Analysis of the PACS craving scale before and after therapy.

Therapy resulted in a significant reduction in craving severity. The median PACS score decreased from 9 (4.0–16.0) at baseline to 4 (1.0–8.0) after treatment, and the total PACS score was significantly reduced (p < 0.001), as shown in **Figure 1**.

Significant differences in changes in alcohol craving were observed across selected demographic groups. Among men, a significant reduction in PACS scores was noted after therapy (p < 0.001), whereas among women the decrease did not reach statistical significance (p = 0.131). A significant reduction was also observed among participants living in urban areas (p < 0.001), while no statistically significant change was found in those living in rural areas (p = 0.214). In economically active individuals, the median PACS score decreased from 10.0 before therapy to 4.0 after therapy (p < 0.001). In economically inactive participants, the median score decreased from 8 to 4 points (p = 0.003). Participants without previous addiction treatment demonstrated a significant reduction in PACS scores, from 8 before therapy to 4 after therapy (p < 0.001). These results are presented in **Table 3** Patients who had treated their addiction 6 years and earlier before therapy scored 5pts vs 0pts after therapy (p=0.004).

**Table 3:** Analysis of the PACS craving scale before and after therapy.

		PACS SUM								p-value Stage I vs Stage II
		STAGE I			p-value	STAGE II			p-value	
		Me	Q1	Q3		Me	Q1	Q3		
Gender	female	8, 00	4, 00	16, 00	0, 523	6, 00	3, 00	9, 00	0, 081	0, 131
	man	9, 00	4, 00	17, 00		4, 00	1, 00	7, 00		<0, 001
Place of origin	urban	9, 00	4, 00	16, 00	0, 344	4, 00	2, 00	8, 00	0, 584	<0, 001
	rural	5, 00	1, 00	16, 00		4, 00	0, 00	8, 00		0, 214
Education	primary	4, 00	2, 00	12, 00	0, 215	2, 00	0, 00	4, 50	0, 154	0, 179
	lower secondary	13, 00	6, 00	22, 00		3, 00	0, 00	7, 00		0, 116
	vocational	8, 00	2, 00	15, 00		4, 50	0, 50	9, 00		0, 005
	secondary	7, 50	3, 50	16, 00		4, 00	0, 50	6, 50		0, 013
	higher	11, 00	5, 50	18, 00		5, 00	4, 00	10, 00		0, 001
Marital status	single	10, 00	4, 00	17, 00	0, 608	4, 00	1, 00	9, 00	0, 577	<0, 001
	in a relationship	8, 00	4, 00	15, 00		4, 00	2, 00	7, 00		<0, 001
Number of children	0	11, 00	5, 00	17, 00	0, 173	0, 00	0, 00	0, 00	0, 959	<0, 001
	1	6, 00	3, 00	15, 00		4, 00	2, 00	5, 00		0, 027
	2	8, 00	4, 00	14, 00		4, 00	2, 00	9, 00		0, 009
	3	7, 00	5, 00	8, 00		4, 50	3, 00	8, 00		0, 285
	5	21, 00	21, 00	21, 00		4, 00	4, 00	4, 00		not enough people have 5 children
Professional activity	yes	10, 00	4, 00	18, 00	0, 451	4, 00	2, 00	7, 00	0, 808	<0, 001
	not	8, 00	4, 00	14, 00		4, 00	1, 00	8, 00		0, 003
Type of work performed	physical	9, 00	4, 00	17, 00	0, 292	4, 00	0, 00	8, 00	0, 497	<0, 001
	white-collar	12, 00	5, 00	19, 00		4, 00	4, 00	7, 00		<0, 001
	retired	5, 00	2, 00	14, 00		4, 00	1, 00	16, 00		0, 575
	on pension	10, 00	7, 00	14, 00		3, 00	2, 00	3, 00		0, 285
Material situation	very good	6, 00	1, 00	14, 00	0, 587	4, 00	2, 00	7, 00	0, 688	0, 612
	good	8, 00	4, 00	15, 00		4, 00	3, 00	8, 00		0, 002
	average	10, 00	5, 00	15, 00		4, 50	1, 00	9, 00		<0, 001
	bad	14, 50	6, 00	18, 00		2, 50	0, 50	6, 00		0, 032
	very bad	5, 00	2, 00	17, 00		4, 00	0, 00	9, 00		0, 225
Housing conditions	very good	8, 00	4, 00	17, 50	0, 179	4, 00	2, 00	5, 00	0, 559	0, 002
	good	8, 00	4, 00	14, 00		4, 00	2, 00	8, 00		0, 003
	average	8, 50	3, 00	15, 00		4, 00	1, 00	8, 00		0, 009
	bad	21, 00	9, 00	26, 50		8, 00	0, 00	15, 00		0, 043
	very bad	17, 00	17, 00	17, 00		0, 00	0, 00	0, 00		not enough people in this group
Previous treatment for alcohol addiction	no	8, 00	3, 00	16, 00	0, 646	4, 00	1, 00	8, 00	0, 413	<0, 001
	yes, 1 to 2 years previously	8, 00	5, 00	16, 00		5, 50	3, 00	10, 50		0, 441
	yes, 3 to 5 years previously	11, 00	7, 00	17, 00		4, 00	0, 00	4, 00		0, 285
	yes, 6 years ago and before	5, 00	5, 00	5, 00		0, 00	0, 00	0, 00		0, 004

Me-median, Q1-first quartile, Q3-third quartile, p-level of statistical significance.

Source: own research.

**Table 4** presents the correlation between the CECS scale and the PACS scale before and after therapy. The analysis showed a positive correlation between feelings of alcohol craving before therapy and suppression of depression (r=0.197, p=0.017), as alcohol craving increases, suppression of depressive feelings increases. After therapy, a negative correlation was found between feelings of alcohol craving and the ability to control anger (r=-0.188, p=0.047) as alcohol craving decreases, the ability to control anger increases.

**Table 4:** Assessment of the relationship between the level of anxiety and anger feelings and the level of alcohol craving severity before and after treatment.

Pair of variables		R	p
PACS scale before	CECS_1_anger BEFORE	0, 006	0, 945
	CECS_2_depression BEFORE	0, 197	0, 017
	CECS_3_anxiety BEFORE	0, 027	0, 750
	SUM OF CECS BEFORE	0, 072	0, 392
	CECS_1_anger AFTER	-0, 072	0, 456
	CECS_2_depression AFTER	0, 071	0, 464
	CECS_3_anxiety AFTER	0, 013	0, 893
	SUM CECS AFTER	0, 009	0, 925
PACS scale after	CECS_1_anger BEFORE	-0, 167	0, 083
	CECS_2_depression BEFORE	0, 024	0, 802
	CECS_3_anxiety BEFORE	-0, 065	0, 500
	TOTAL CECS BEFORE	-0, 098	0, 312
	CECS_1_anger AFTER	-0, 188	0, 047
	CECS_2_depression AFTER	-0, 052	0, 583
	CECS_3_anxiety AFTER	0, 001	0, 992
	CECS AFTER	-0, 094	0, 323

R-correlation coefficient, p-level of statistical significance.

Analysis of the Control of Emotions Scale (CECS) before and after therapy was carried out according to selected demographic data. The results are presented in **Table 5**. After therapy, statistically significant differences were observed in terms of occupational activity, with those working after therapy scoring significantly lower on the CECS scale (p=0.012), which translates into greater control over negative emotions. Retired individuals had the highest median emotion control scale scores (p=0.014). In the male group, a significant decrease in scale scores of 54.0 vs 49.5 (p<0.001) was observed before and after therapy. Among physical workers, the study showed a significant decrease in scores before therapy 55.0 vs after therapy 48.0 (p<0.001).

**Table 5:** Analysis of the emotion control scale (CECS) before and after therapy. .

		CECS SUM								p-value Stage I vs Stage II
		STAGE I			p-value	STAGE II			p - value	
		Median	Lower	Upper		Median	Lower	Upper		
Gender	female	50, 50	46, 00	63, 00	0, 121	47, 00	38, 00	55, 00	0, 109	0, 062
	male	54, 00	49, 00	62, 50		49, 50	42, 00	58, 00		<0, 001
Place of origin	urban	53, 00	47, 00	63, 00	0, 887	49, 00	41, 00	57, 00	0, 793	<0, 001
	rural	53, 00	47, 00	60, 00		48, 50	36, 00	53, 00		0, 032
Education	primary	56, 00	51, 50	58, 50	0, 606	51, 00	43, 00	58, 50	0, 759	0, 285
	lower secondary	54, 50	49, 00	61, 00		45, 50	37, 00	55, 00		0, 249
	vocational	58, 50	48, 00	67, 00		53, 00	41, 00	60, 00		0, 006
	secondary	53, 00	47, 00	61, 00		47, 00	39, 50	57, 00		0, 033
	higher	52, 00	47, 00	57, 00		49, 00	42, 00	54, 00		0, 257
Marital status	single	55, 00	49, 00	63, 00	0, 303	47, 00	42, 00	55, 00	0, 981	0, 003
	in a relationship	53, 00	47, 00	63, 00		49, 00	40, 00	58, 00		0, 008
Number of children	0	53, 50	50, 00	62, 00	0, 283	46, 00	38, 00	50, 00	0, 147	<0, 001
	1	52, 00	41, 00	61, 00		51, 50	44, 00	57, 00		0, 829
	2	54, 50	48, 00	67, 00		53, 00	43, 00	59, 00		0, 037
	3	49, 00	46, 50	58, 50		54, 00	48, 00	56, 00		0, 829
	5	42, 00	42, 00	42, 00		41, 00	41, 00	41, 00		not enough people have 5 children
Professional activity	yes	53, 00	47, 00	61, 50	0, 145	46, 50	40, 00	54, 00	0, 012	<0, 001
	not	55, 00	49, 00	65, 00		53, 00	46, 00	61, 00		0, 151
Type of work performed	physical	55, 00	48, 00	64, 00	0, 296	48, 00	39, 00	56, 00	0, 014	<0, 001
	white-collar	50, 00	47, 00	56, 00		46, 50	42, 00	52, 00		0, 080
	retired	55, 00	48, 00	61, 00		60, 00	54, 00	65, 00		0, 214
	on pension	61, 00	47, 00	65, 00		55, 00	49, 00	79, 00		0, 109
Material situation	very good	53, 00	47, 00	66, 00	0, 897	39, 00	37, 00	56, 00	0, 417	0, 116
	good	53, 00	47, 00	62, 00		50, 50	43, 00	56, 50		0, 102
	average	54, 00	48, 50	63, 50		49, 00	40, 00	58, 00		0, 018
	bad	51, 50	46, 00	63, 00		46, 50	42, 50	52, 50		0, 158
	very bad	54, 00	49, 00	58, 00		41, 50	34, 00	50, 00		0, 079
Housing conditions	very good	52, 00	45, 00	69, 00	0, 561	51, 50	44, 50	56, 00	0, 616	0, 097
	good	53, 00	48, 00	62, 00		49, 00	40, 50	58, 00		0, 015
	average	55, 00	48, 00	63, 00		47, 00	43, 00	56, 00		0, 032
	bad	58, 50	53, 00	61, 00		37, 00	36, 00	42, 00		0, 273
	very bad	70, 00	70, 00	70, 00		54, 00	54, 00	54, 00		too few people
Previous treatment for alcohol addiction	no	53, 00	47, 00	62, 00	0, 825	50, 00	41, 00	57, 50	0, 458	0, 005
	yes, 1 to 2 years previously	52, 50	45, 00	65, 00		44, 00	39, 00	56, 00		0, 255
	yes, 3 to 5 years previously	55, 00	51, 00	74, 50		46, 00	46, 00	46, 00		0, 068
	yes, 6 years ago and earlier	56, 00	49, 00	58, 00		47, 00	45, 00	58, 00		0, 078

Me-median, Q1-first quartile, Q3-third quartile, p-level of statistical significance.

Source: Own research.

The study also showed that after therapy, alcohol craving increased in 27 (17.31%) patients. This result can be interpreted to mean that the patients’ awareness of recognising uncharacteristic craving symptoms increased during therapy.

Interesting correlations are observed with regard to the severity of alcohol craving and the management of adverse emotions in the post-therapy period. In 23 patients, the overall level of suppression of unpleasant emotions also increased with the severity of alcohol craving, and 39 patients reported greater openness in expressing them. This interesting correlation may be explained by patients’ fears of completing primary therapy and returning to family and work life.

## 4 Discussion

Alcohol craving is indeed linked to the concept of alcohol relapse; as a symptom of a strong, irresistible desire to drink alcohol, it is one of the most common warning signs of impending relapse after a period of abstinence. The sight of a favourite drink, alcohol advertising or waking up after an alcoholic dream can trigger the desire for alcohol. High risk factors for relapse also include experiencing unpleasant feelings such as boredom, sadness, anxiety, anger, depression, guilt or loneliness, but also pleasant situations, which can include professional and personal success or falling in love. Conflicts, the influence of the environment, especially suggestions or persuasion to drink alcohol, somatic complaints such as headaches, toothaches or sleep problems are also important. Controlled drinking attempts due to the belief that: “this time it will work”, “I can already”, “after such a time nothing will surely happen” (8, 25). The specific nature of the alcohol disease makes both patients and professionals interested in the effectiveness of treatment and relapse prevention. However, studies in this area show that 35% of patients treated for alcohol dependence are unable to maintain abstinence in the first 2 weeks after completing therapy and 58% experience relapse within 3 months. Maintaining complete abstinence within 1 year of completing treatment is reported by 19% of outpatients and 35% of those in 24-hour units (26, 27). Analyses have also been conducted on which therapeutic strand produces the best results in the treatment of alcohol dependence. A comparative analysis in a review by Robert Modrzyński found comparable effectiveness of the available methods (28). The literature reports a significant relationship between anxiety disorders and the risk of alcohol relapse and sustained drinking (29). Anxiety and alcohol abuse can be mutually inducing. The effects of ethanol can reduce unpleasant anxiety symptoms which provides positive reinforcement, which may consequently increase further drinking. In contrast, drinking alcohol can increase anxiety and worry (30, 31). The same is true for depressive disorders, which are most strongly associated with a high risk of relapse. However, research shows that depressive states are not necessarily linked to treatment failure in alcohol-dependent patients. Some patients with comorbid depressive symptoms achieved longer periods of abstinence than patients without such symptoms (32–35). The relationship between alcohol abuse and feelings of anger, according to studies, can be induced by environmental factors such as domestic violence or functioning in destructive family or professional relationships (20). Environmental and occupational factors are significant for the findings described in this article due to the fact that 88.8% of the respondents are of working age and 59.2% have children. The Children’s Ombudsman, on the basis of the cases he analysed, indicated that the most common reasons for the interference of services in the functioning of families, including family courts regarding parental authority were: alcoholic illness of parents – 52%, neglect of care and upbringing, including health and hygiene – 39%, violence, including physical, psychological and sexual violence – 36%, difficult financial situation of the family – 21% of the analysed cases, lack of adequate supervision by parents – 18%, mental disorders of parents – 12% and leaving the child in the care of another family member or in hospital – 27%. At the same time, he stressed that the problems and factors listed did not occur alone. In 88% of the cases studied, at least three of the problems listed were present together (36). The above circumstances are the most common reasons for securing a child in foster care. In addition, the experience of childhood trauma by parents of children placed in foster care points directly to a link between these negative experiences and the consequences in their adult life, including depression, suicide attempts, addiction tendencies, relationship difficulties and being a perpetrator or victim of violence (37). Alcohol dependence in the context of work can lead to consequences such as absenteeism, decreased productivity, presenteeism and difficulties in maintaining relationships with colleagues, as well as loss of employment. Moreover, the type of work performed, the conditions and hours of work, the form of employment, the burden of responsibilities and occupational stress can all be contributing factors to alcohol misuse (38–40). Many people who decide to seek treatment for alcohol dependence are in a difficult life situation. Alcohol consumption was their previous method of regulating their depressive state or emotions such as anxiety and anger. After treatment in the day ward, most of them return to their previous activities. The dismantling of the psychological mechanisms of addiction in such individuals can exacerbate the stress of returning to work and the lack of understanding from friends, acquaintances and family. This results in a kind of vicious circle in addiction psychotherapy and, consequently, a return to drinking (28). Studies show that undergoing primary therapy in a day ward setting results in a significant decrease in perceived craving for alcohol on the PACS scale (14, 23), as well as a lower overall index denoting the individual’s subjective belief related to the ability to control responses in response to experiencing unpleasant emotions based on the CECS scale (22). The persistent tendency to inhibit unpleasant emotions becomes the substrate for many neurotic and psychosomatic disorders, hence the ability to name unpleasant emotions and express them is an important element in alcohol dependence psychotherapy (41). Recognising the symptoms of alcohol craving, being able to cope with these feelings, and naming and expressing adverse emotions in a socially acceptable way brings about changes in the daily functioning of the addicted person, promotes the improvement or restoration of family, social and professional relationships, and brings about improvements in health and quality of life. The patient, thanks to the change made, recovers partially or completely his or her resources, understood as valued persons, things or qualities of particular importance, among others: family, health, material goods, work, security, competence. Depending on the rank he or she gives to the change and his or her confidence in his or her ability to make it happen, he or she finds the motivation to maintain abstinence. This approach is confirmed by work in the spirit of motivational therapy (42, 43). In contrast, the base of recovered resources, according to Hobfoll’s Conservation of Resources Theory (COR), causes people to strive to obtain, maintain and protect what is valuable to them (44). According to COR, what most threatens a person’s proper functioning is the sudden loss of resources, causing them to experience heightened feelings of discomfort and stress, at which point pre-abstinence defence mechanisms may be triggered, causing alcohol relapse. According to this approach, in therapeutic work with alcohol-dependent patients, it is not so much important to focus on the percentage evaluation of the effectiveness of the therapy, but on achieving lasting change. The goal set by the patient, the awareness of the discrepancy between the current state and the one he or she is striving for, and the recovery of lost or accumulation of new values and resources are motivating factors for sustaining lasting change. Patients entering therapy in a day ward have diverse needs, values and resources, so it is important to tailor therapy to the individual needs and abilities of patients. For this purpose, addiction therapists do not focus on one particular stream, but combine elements of different therapeutic approaches using an integrative model by adapting working methods and techniques to the specific needs and problems of the patient (45).

## 5 Conclusions

Alcohol dependence therapy in the form of a day ward significantly reduces the severity of craving, strengthens the conscious recognition of the symptoms of alcohol craving and equips with coping skills, and increases the ability to resist the temptation to use alcohol when there is an opportunity to drink. This change is particularly noticeable in men and in people who have already received therapeutic services in this area. Completing Basic Therapy improves competence in recognising and naming negative emotions, as well as talking about and expressing them in an acceptable way. The most significant factor influencing the level of expression of negative emotions is work activity.

### 5.1 What this paper adds

For patients, the results of the study provide evidence that participation in primary therapy in the form of a day ward offers the opportunity to regain control over life and improve family relationships and function better in professional and social life. The research described does not guarantee the effectiveness of the therapy, but raises awareness of important changes that, if consolidated, can influence the avoidance of relapse and longer and more satisfying abstinence.

For therapists, the article provides measurable confirmation in terms of the impact of therapy on reducing feelings of craving for alcohol and lowering levels of suppression of negative emotions. This knowledge can inspire the search for methods and tools to strengthen patients’ motivation to recover and to build their container of resources and values, making sobriety a goal in itself.

For employers, the results obtained are a pointer to the rationale for introducing an alcohol prevention strategy in the workplace, one element of which may be to consider the participation of employees with alcohol use problems in primary therapy as part of their sick leave.

For policy makers, this article confirms that funding alcohol dependence treatment is an investment with tangible results and a reduction in the social and economic costs of omission.

### 5.2 Limitations

A limitation is the lack of longitudinal studies in previous years, which makes it impossible to observe and compare the current study with previous studies in terms of the changes taking place. Limitations also include the process of monitoring the abstinence of patients included in the study, which is important in terms of determining the sustainability of change.

## Data Availability

The raw data supporting the conclusions of this article will be made available by the authors, without undue reservation.
